# CO Adsorption on a Single‐Atom Catalyst Stably Embedded in Graphene

**DOI:** 10.1002/anie.202421757

**Published:** 2025-01-21

**Authors:** Daniele Perilli, Valeria Chesnyak, Aldo Ugolotti, Mirco Panighel, Stefano Vigneri, Francesco Armillotta, Pardis Naderasli, Matus Stredansky, Monika Schied, Paolo Lacovig, Silvano Lizzit, Cinzia Cepek, Giovanni Comelli, Harald Brune, Cristina Africh, Cristiana Di Valentin

**Affiliations:** ^1^ Department of Materials Science University of Milano-Bicocca via R. Cozzi 55 I-20125 Milano Italy; ^2^ Physics Department University of Trieste via A. Valerio 2 Trieste 34127 Italy; ^3^ CNR – Istituto Officina dei Materiali (IOM), Trieste Strada Statale 14, km 163.5 34149 Trieste Italy; ^4^ Institute of Physics Ecole Polytechnique Fédérale de Lausanne (EPFL) Station 3 CH-1015 Lausanne Switzerland.; ^5^ Elettra – Sincrotrone Trieste S.C.p.A., s.s. 14 km 163.5 34149 Trieste Italy; ^6^ Present address: School of Chemical, Biological, and Environmental Engineering Oregon State University Corvallis OR 97331 United States and Physical and Computational Sciences Directorate and Institute for Integrated Catalysis Pacific Northwest National Laboratory Richland, WA 99354 United States.; ^7^ Present address: Scanning Probe Microscopy Laboratory Department of Physics and Materials Science University of Luxembourg Luxembourg City L-1511 Luxembourg; ^8^ Present address: School of Chemistry University of Birmingham Edgbaston University Rd W Birmingham B15 2TT United Kingdom; ^9^ Present address: CNR – Istituto Officina dei Materiali (IOM), Trieste, Strada Statale 14, km 163.5 34149 Trieste Italy

**Keywords:** Carbon Monoxide, Density Functional Theory Calculations, Graphene, Scanning Tunnelling Microscopy, Single Atom Catalysts, Temperature Programmed Desorption

## Abstract

Confined single metal atoms in graphene‐based materials have proven to be excellent catalysts for several reactions and promising gas sensing systems. However, whether the chemical activity arises from the specific type of metal atom or is a direct consequence of the confinement itself remains unclear.

## Graphical abstract

This study explores the chemical activity of isolated metal atoms confined in graphene on Ni(111), using carbon monoxide as a probe molecule. At room temperature, CO stably adsorbs on Co but not on Ni. Electronic descriptors clarify this behavior, offering valuable insights for designing graphene‐based single‐atom catalysts.

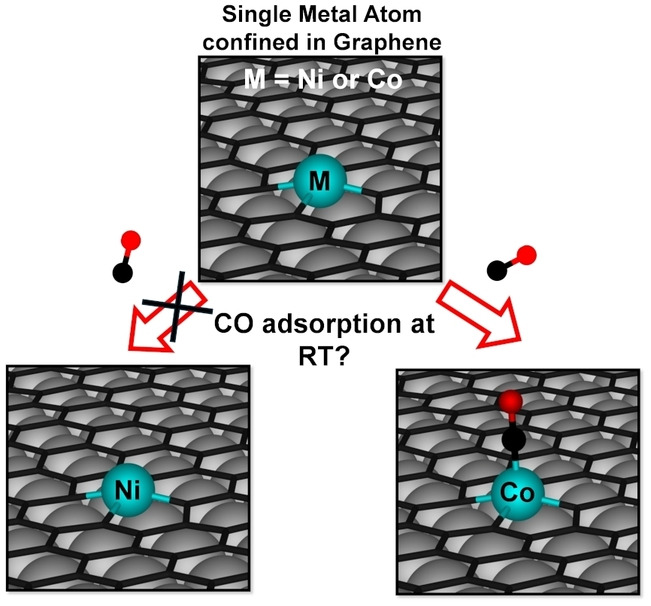

In this work, through a combined density functional theory (DFT) and experimental surface science study, we address this question by investigating Co and Ni single atoms embedded in graphene (Gr) on a Ni(111) support. These two single atom catalysts (SACs) exhibit opposite behavior toward carbon monoxide (CO) gas molecules: at room temperature, CO binds stably to Co, whereas it does not bind to Ni. We rationalize this difference by the energy position of the trapped metal d_xz_ and d_yz_ states involved in π backdonation to CO: while for Co, these states lie at the Fermi level, for Ni they are located deep below it.

This conclusion is corroborated by a proof‐of‐concept experiment, where a Gr/Ni(111) sample containing both stable Ni and Co single atoms was exposed to a CO partial pressure of 5 ⋅ 10^−7^ mbar. Scanning tunnelling microscopy (STM), X‐ray photoelectron spectroscopy (XPS), and temperature programmed desorption (TPD) measurements confirm the selective adsorption of CO on Co at RT.

## Introduction

One of the biggest challenges facing our society is the transition towards greener and more sustainable manufacturing processes, where catalysts play a pivotal role.[^1^, ^2^] The development of highly efficient materials, featuring low amounts of precious metals and ensuring cost‐effectiveness, is a requirement more urgent than ever.^[3]^ In this regard, single‐atom catalysts (SACs) are emerging as promising solutions for the next generation of catalysts.^[4]^ By dispersing individual transition metal (M) atoms onto a suitable support material, SACs offer the selectivity and activity of homogeneous systems, along with the stability and recoverability typical of heterogeneous systems.^[5]^

Beyond catalysis, there is a recent growing attention for SACs as promising sites for efficient gas capturing and sensing, which has become a new area of active research with important implications for health care and environmental protection.[^6^, ^7^]

A critical aspect in SAC design, independently of the final application, is the choice of a substrate capable of effectively stabilizing and preventing the aggregation of isolated metal atoms into larger particles.^[5]^ Beyond mere stabilization, the substrate may also strongly influence the electronic properties of the trapped metal atom, similar to homogeneous systems where ligands shape the coordination sphere.^[8]^

In this scenario, graphene (Gr) is considered to be a promising platform for metal trapping, thanks to its large surface‐to‐volume ratio, conductivity, and its capability to stabilize metals through robust covalent bonds,^[9]^ limiting the mobility and the possibility of aggregation that is observed for SACs adatoms on traditional metal oxide supports.[^10^, ^11^, ^12^] In 2013, Sun et al.^[13]^ succeeded in preparing and testing the first Gr‐based SAC, where isolated Pt atoms were effectively anchored onto Gr nanosheets, boosting the catalytic activity for the methanol oxidation reaction (MOR). Subsequent experimental studies have focused on refining preparation methods and characterization techniques to gain a deeper insight into the structure of the active metal site.[^14^, ^15^]

Regarding the catalytic activity of Gr‐based SAC systems, remarkable results have been obtained for benzene oxidation,[^16^, ^17^] CH_4_ oxidation,^[18]^ and selective hydrogenation of acetylene^[19]^ in thermally activated processes, as well as oxygen reduction reaction (ORR),[^20^, ^21^] CO/CO_2_ reduction reaction (CRR),[^22^, ^23^, ^24^] and water splitting[^25^, ^26^] in electrocatalytic processes. As for sensing, Zhou et al. reported superior NO sensing performances by isolated Ni atoms in a Gr‐based matrix with respect to commonly used Ni‐based nanomaterials.^[27]^

In this context, experimental time‐ and space‐resolved techniques are often combined with computational methods to disclose the structure of the metal active site and elucidate its chemical activity.[^28^, ^29^] In most cases, the activity of SACs is rationalized from an electronic point of view.^[30]^ Indeed, the confined metal atom displays localized atomic states near the Fermi level of the host system. These states, whether filled or empty, may favor oxidative or reductive chemical processes, respectively.

The questions that arise are: (i) whether the chemical activity of SACs is mainly dictated by the type of metal atom trapped, or by the metal confinement in the bidimensional Gr layer, implying that the nature of the metal itself is less relevant and (ii) whether the underlying metal support plays a role. Answering these questions is crucial for designing smart SAC‐based systems, as it requires an understanding of all factors contributing to the system‘s reactivity, identifying those with greater significance, and thereby appropriately guiding the material preparation.

Following this stream, in our recent work,^[31]^ we successfully created and thoroughly characterized a Gr‐based system where individual Co atoms are trapped within the Gr network. Employing a bottom‐up strategy via chemical vapor deposition (CVD) growth on a Ni(111) substrate, we synthesized a high‐quality Gr layer with a controlled amount of incorporated Co atoms, leading to a precisely defined morphology. Additionally, besides Co, Gr synthesized via CVD on Ni surfaces exhibits intrinsic doping by Ni atoms.^[32]^ This results from surface Ni adatoms, which are highly mobile at CVD temperatures,^[33]^ and interact with the growing Gr edges, eventually remaining trapped within the layer during the growth process.^[26]^

The unique combination of Co and Ni sites within the same Gr layer, each exhibiting the same geometrical structure, provides ideal conditions for directly comparing the chemical activity of SACs between two different metal elements.

In this study, we aim at uncovering whether the chemical activity of SACs is primarily determined by the chemical nature of the specific metal or rather by its confinement within Gr. To address this issue, we performed density functional theory (DFT) calculations, investigating the adsorption behavior of carbon monoxide (CO), as a sensitive probe molecule of the electron density on a metal center,^[34]^ on Ni and Co atoms embedded within Gr supported on Ni(111) (M@Gr/Ni, where M=Co or Ni). CO adsorption on the SAC site is not only the first key step in both oxidation to CO_2_ or reduction to sustainable chemicals, which are the major CO‐involving industrial processes, but also for CO capturing and detection with impact on human health and environment. Unveiling the SAC‐CO electronic interplay, which dictates the details of the adsorption mechanism, will allow an optimal control for the system design.

We found that, despite the fact that CO adsorbs in similar configurations on both Co and Ni, the two systems behave differently. Specifically, Co, unlike Ni, stably binds CO at room temperature (RT). Our theoretical findings are further validated through proof‐of‐concept experiments, where the M@Gr/Ni system was exposed to a CO atmosphere at 5 ⋅ 10^−7^ mbar pressure, and the adsorption behavior was examined using microscopic and spectroscopic techniques.

## Results and Discussion

**Figure 1:**
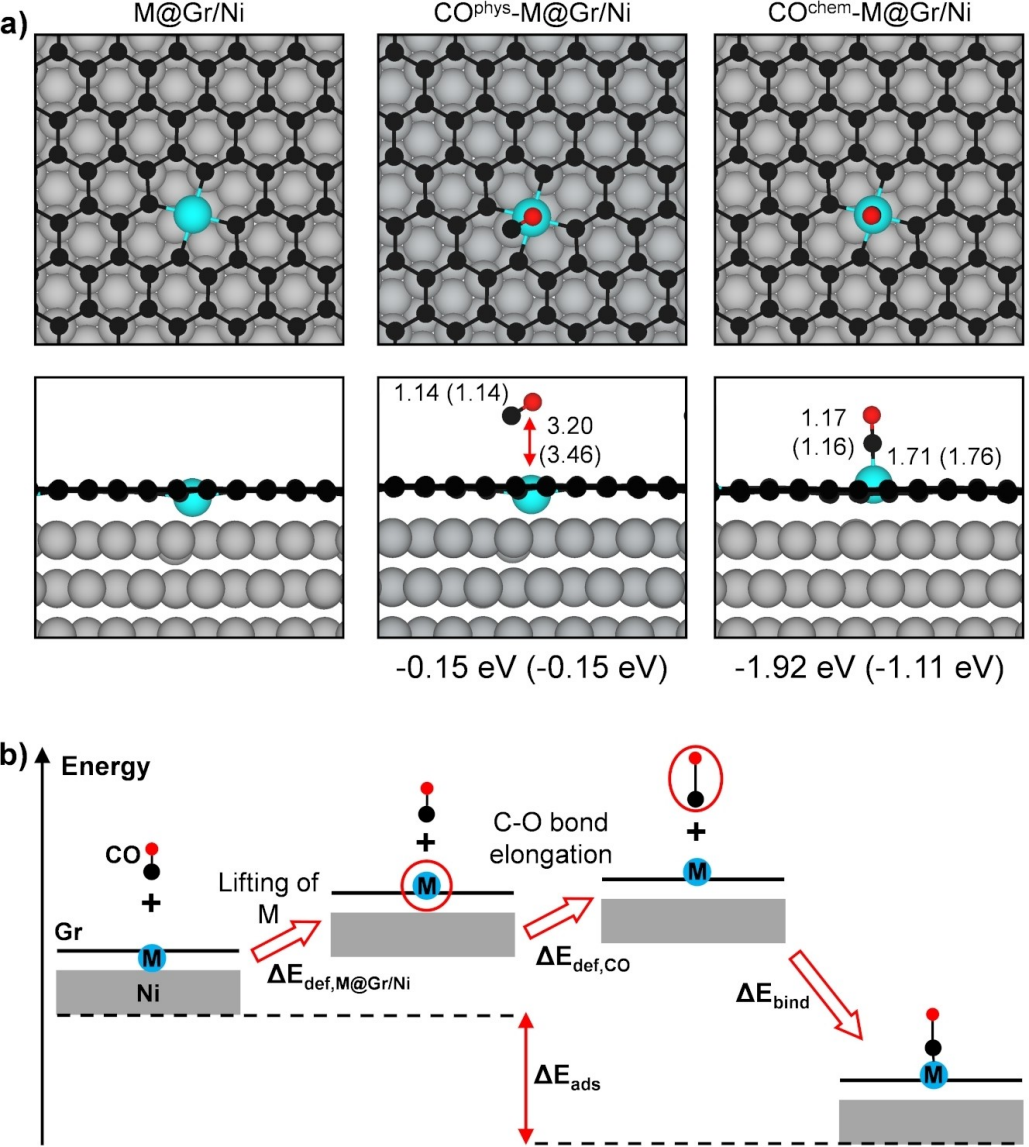
a) Top and side views of the optimized geometries with and without a CO molecule adsorbed on M@Gr/Ni. Color coding: Ni substrate atoms are grey, C atoms in Gr are black, confined M atoms are cyan, and C and O atoms in CO are black and red, respectively. Adsorption energies (ΔE_ads_) and M–CO, C−O, and M−C distances (in Å) for Co (Ni) are reported below each configuration. b) Schematic representation of the energy decomposition analysis for the energy contribution of deformation (positive, ΔE_def_) and of binding (negative, ΔE_bind_) to the adsorption energy (negative, ΔE_ads_) of CO on the M@Gr/Ni surface. ΔE_def_ consists of two contributions: the deformation of the M@Gr/Ni system (ΔE_def,M@Gr/Ni_) and the deformation of CO (ΔE_def,CO_). The energy values for these contributions are listed in Table 1.

We begin by investigating the reactivity of M@Gr/Ni using a model developed in our previous work, in agreement with experimental STM observations,^[25]^ where the most common configuration corresponds to either a Co or a Ni atom trapped within a C double vacancy site of Gr (Figure 1a). Upon the introduction of CO, we identify two distinct minimum energy structures: in one case, the molecule weakly physisorbs about 3 Å above the Gr plane on top of the trapped metal (CO^phys^‐M@Gr/Ni in Figure 1a), while in the other, it chemisorbs (see CO^chem^‐M@Gr/Ni in Figure 1a), coordinating with the metal via the C atom of the molecule in a terminal mode. Despite the similar geometrical structure of chemisorbed CO on both metals, there is a notable difference in energetics: CO adsorbs more strongly on Co (ΔE_ads_ = −1.92 eV) than on Ni (ΔE_ads_ = −1.11 eV).

To verify whether this difference truly reflects a distinct M–CO interaction, we employed an energy decomposition scheme,^[35]^ shown in Figure 1b, to determine the energy contribution of structural deformations (positive, ΔE_def_) and of binding (negative, ΔE_bind_=E_CO_^chem^_‐M@Gr/Ni_ – (E_CO,unrelax_ + E_M@Gr/Ni,unrelax_)) to the adsorption energy (ΔE_ads_=E_CO_^chem^_‐M@Gr/Ni_ – (E_CO,relax_ + E_M@Gr/Ni, relax_)) of CO on M@Gr/Ni (with M=Co or Ni). As shown in Table 1, the deformation costs are similar for both systems (approximately 0.6 eV), indicating that CO indeed binds (ΔE_bind_) more strongly to Co compared to Ni.

**Table 1:** Calculated Electronic (ΔE) and Gibbs Free (ΔG_ads_) energy values for CO physisorption (*Phys*) and chemisorption (*Chem*) on M@Gr/Ni. For the chemisorbed case, energy values obtained from the energy decomposition analysis, as illustrated in Figure 1b, are provided. These include contributions from the deformation of M@Gr/Ni (ΔE_def,M@Gr/Ni_) and CO (ΔE_def,CO_), as well as the binding energy (ΔE_bind_), influencing the adsorption energy (ΔE_ads_) of CO on the M@Gr/Ni surface. The ΔG_ads_ values are calculated as G_CO‐M@Gr/Ni_ ‐ G_M@Gr/Ni_ ‐ G_CO_, where the reference systems are optimized in their isolated states. The Gibbs free energy (G) values are computed at 298 K, with further details on the calculation methods provided in the Computational Details section of the SI. All energy values are expressed in eV.

	*Phys*		*Chem*				
	ΔE_ads_	ΔG_ads_	ΔE_def,M@Gr/Ni_	ΔE_def,CO_	ΔE_bind_	ΔE_ads_	ΔG_ads_
Co@Gr/Ni	−0.15	+0.05	+0.59	+0.04	−2.56	−1.92	−1.34
Ni@Gr/Ni	−0.15	+0.05	+0.54	+0.02	−1.67	−1.11	−0.56

**Figure 2:**
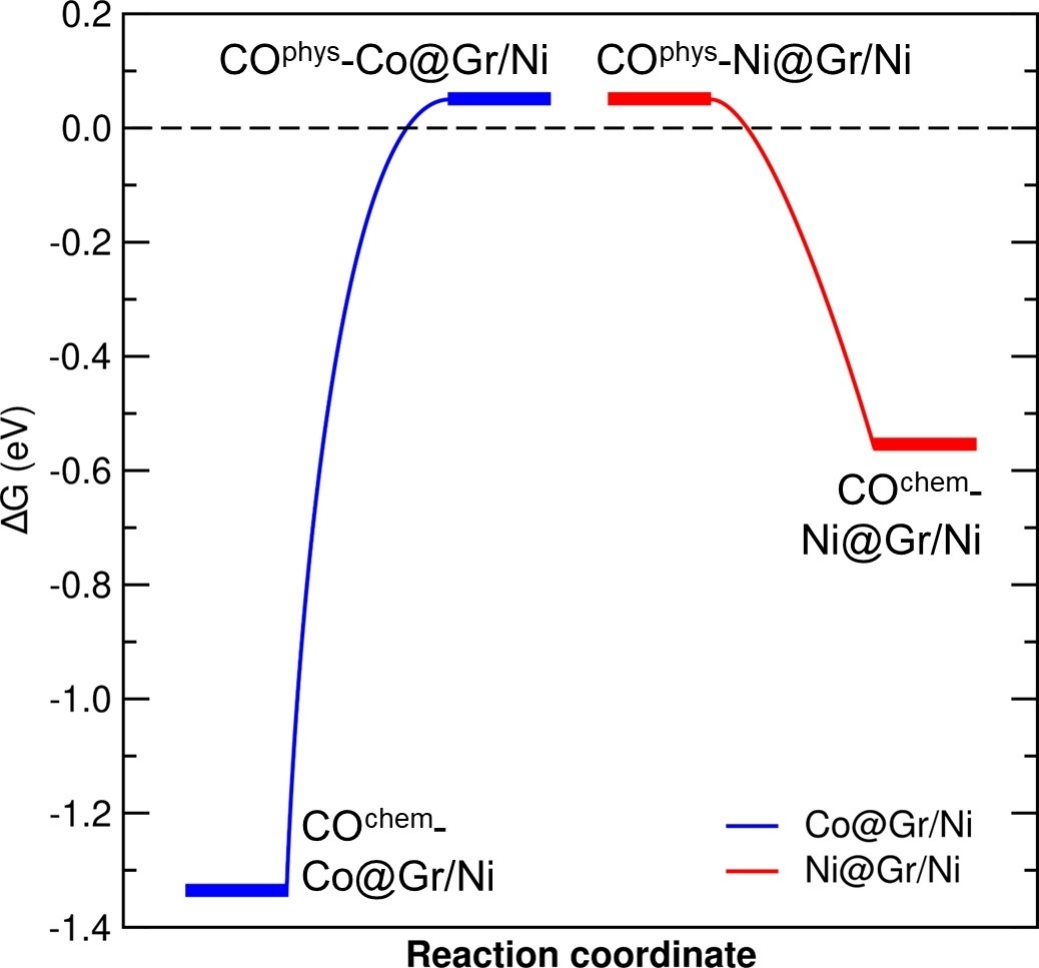
Variation in Gibbs free energy (ΔG) at 298 K for CO chemisorption on the metal within Gr/Ni. Blue and red lines represent Co and Ni cases, respectively. The energy profile, connecting the physisorbed and chemisorbed configurations, was obtained using a climbing‐image nudged elastic band (CI‐NEB) calculation with 5 intermediate images. Ball‐and‐stick models of the CI‐NEB images are shown in Figure S1.

Although CO binds more strongly to Co than Ni, both metals exhibit a strong interaction. However, two factors should be considered. Firstly, when a gas‐phase molecule is adsorbed, its rotational and translational degrees of freedom are quenched, leading to an energy cost in terms of entropy loss. Secondly, there may be a kinetic barrier preventing molecule chemisorption, making the stable minimum inaccessible at RT. Therefore, we computed the Gibbs free energy (ΔG) profile for CO chemisorption on Ni@Gr/Ni (red curve) and Co@Gr/Ni (blue curve), resulting in barrierless processes, as shown in Figure 2.

On this basis, we can deduce the following scenario. Assuming an equal number and distribution of Ni and Co sites in Gr, a CO molecule will have an equal chance of encountering either metal. If Ni comes first, CO will initially physisorb, followed by chemisorption. This adsorption free energy (ΔG_ads_ = −0.56 eV) is less negative compared to the corresponding electronic energy difference value (ΔE_ads_ = −1.11 eV), due to the entropic expense associated with adsorbing the molecule. Notably, the adsorption step, based on a climbing‐image nudged elastic band (CI‐NEB) calculation, results in being barrierless from a kinetic point of view. Consequently, CO desorption from the Ni site remains highly probable, given that an adsorption free energy of 0.56 eV gives a maximum desorption rate at T >192 K, according to Redhead approximation^[36]^ using a preexponential factor of 10^13^ Hz and a heating rate of 1 K/s. Conversely, if CO interacts with a Co site (blue curve in Figure 2), physisorption will be followed by a fast chemisorption (barrierless), resulting in ΔG_ads_= −1.34 eV, a value sufficiently negative to ensure stable adsorption at RT and up to T <448 K. This implies that the two metals, despite their similar geometric structure when bound to CO, behave differently: Ni is not capable to stably bind CO at RT, while Co is. The different behavior stems from their different strengths of interaction with CO. This raises the question: why does Co interact more strongly?

**Figure 3:**
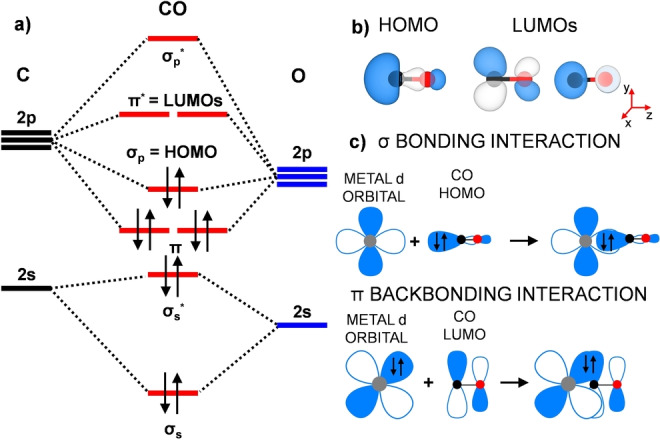
a) Molecular orbitals diagram of CO in the gas‐phase. b) 3D plots for HOMO and LUMOs of CO. Atom color coding: C in black and O in red. The isosurface value is set to 1×10^−2^ e^−^/bohr^3^. c) Schematic representation of the σ and π interactions, illustrating the orbitals involved in the interaction.

In coordination chemistry, the bond between CO and a metal center can arise from two distinct contributions: (i) a σ interaction, which entails a mixing between the HOMO of CO (Figure 3a,b), mostly located at the carbon atom, and the empty metal d‐states, resulting in an electron charge donation from CO to M (Figure 3c); and (ii) a π interaction (Figure 3c) between the two doubly degenerate CO π* LUMOs (Figure 3a,b) and the occupied metal d states with proper symmetry, leading to a charge transfer from the metal to CO.^[37]^ Typically, the metal‐to‐CO π backdonation is much more important than the CO‐to‐metal σ donation.[^38^, ^39^] Therefore, the strength of the M–CO bond will primarily depend on the extent of the former.

Since CO LUMOs exhibit an antibonding character and are filled via π backdonation, one method to assess the strength of the M–CO bond is by examining the C−O distance and the frequency of CO bond stretching. In simpler terms, stronger π backbonding results in a more robust M–CO interaction, causing a larger C−O distance and a decrease in the frequency of CO stretching, known as the red shift effect. This aspect, particularly the red shift, is highly sensitive and commonly utilized in vibrational spectroscopy to evaluate the M–CO interaction.^[40]^ For this reason, as further evidence of the stronger Co‐CO interaction compared to Ni‐CO, we computed the stretching frequencies of the adsorbed CO. We obtain a CO stretching frequency of 1964 cm^−1^ when binding to Co, which is 32 cm^−1^ lower than when binding to Ni (1996 cm^−1^). Accordingly, the C−O distance is also slightly larger in the case of Co‐CO than Ni‐CO (1.17 Å vs 1.16 Å, respectively), confirming the stronger interaction with Co compared to Ni. We note that CO adsorption on both metals results in a redshift in the CO stretching frequency due to the π backbonding, since the computed gas‐phase CO value is 2131 cm^−1^.

Following the reasoning above, one would expect that metals with low oxidation states, i.e. more electron‐rich, will form stronger bonds with CO. However, according to the computed atomic charges, both Ni and Co trapped within Gr acquire the same formal oxidation state of +1, as shown in Table S1 and discussed in our recent work.^[31]^ Our calculations reveal that this extra electron is transferred to Gr, increasing its negative charge by −0.01 *e* per C atom and n‐type doping, as shown in Table S1. In contrast, no charge variation is observed for the Ni(111) substrate.

**Figure 4:**
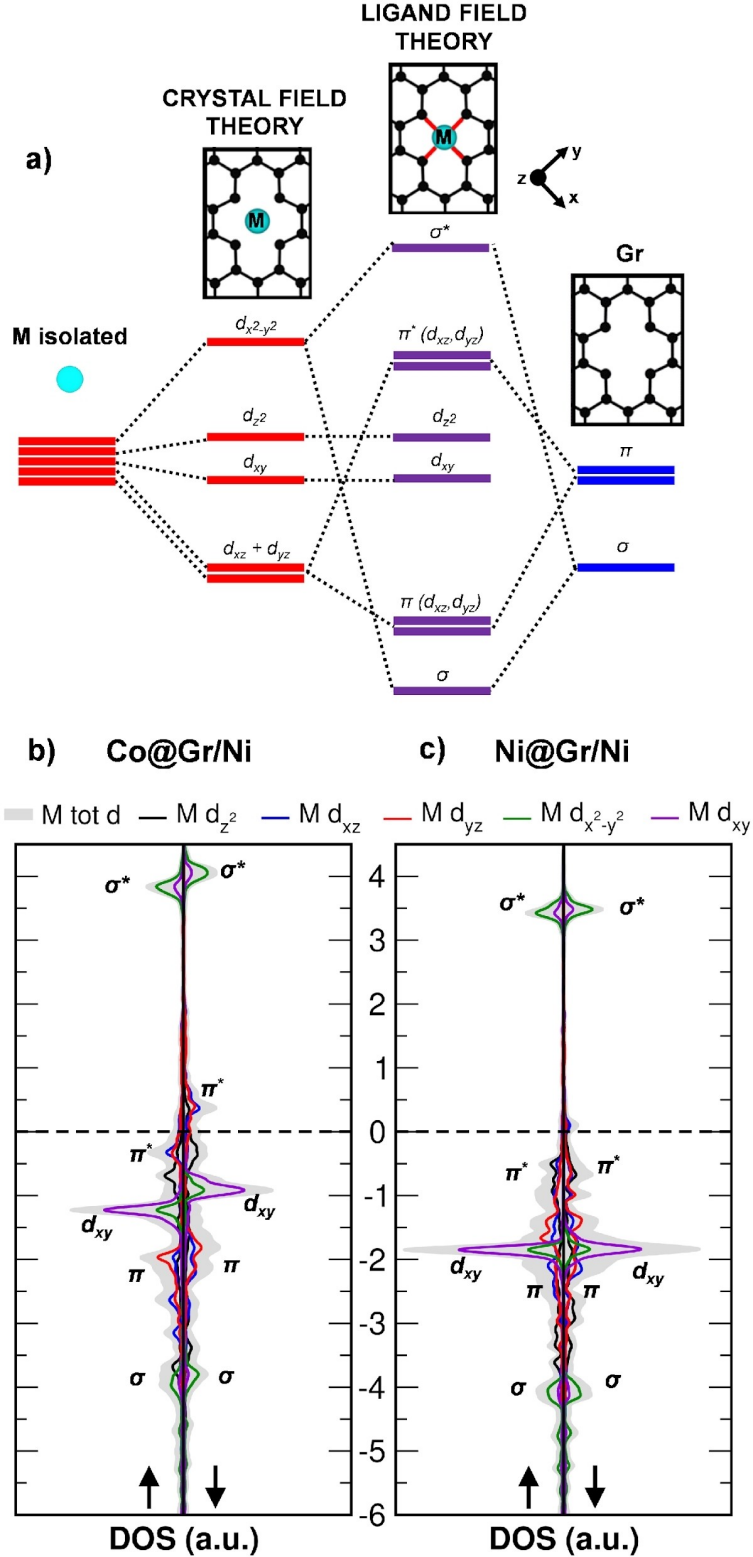
a) Schematic representation of d‐orbital splitting in a square planar D_4h_ coordination environment with free‐standing Gr. From left to right: initially degenerate d‐orbitals of a free metal atom, subsequent crystal field splitting of d‐orbitals resulting from electrostatic interaction (in red), and formation of a new set (in violet) of bonding, nonbonding, and antibonding orbitals (ligand field splitting) through the mixing of d‐orbitals with the σ and π states (in blue) of Gr. The top panels show a ball‐and‐stick model of the metal site, along with the reference coordinate system (in black) used fir the d orbitals. b,c) Projected density of states (PDOS) on metal d orbitals for Co@Gr/Ni (b) and Ni@Gr/Ni (c) systems. The Fermi level is scaled to zero and is indicated by a dashed line. Up and down arrows indicate spin up and down projections, respectively.

Consequently, we must pay attention to what happens to the d electrons that remain on the metal atoms trapped in Gr.^[31]^ If we consider only the coordination with the four C atoms of Gr, neglecting the underlying metal substrate, based on the crystal field theory, the five d‐orbitals of a transition metal atom with square‐planar coordination in D_4h_ symmetry split as follows: the doubly degenerate d_xz_ and d_yz_ are the most stable, followed by d_xy_, d_z2_, and d_x2‐y2_ (refer to the scheme in Figure 4a). However, the covalent interaction with neighboring C atoms suggests that the splitting of metal d states is better explained using ligand field theory, as proposed in our previous works.[^41^, ^42^, ^43^] The in‐plane interaction involves the mixing of metal d_x2‐y2_ and the σ states of the four neighboring C atoms, forming bonding σ and antibonding σ* states (Figure 4a). Conversely, the out‐of‐plane interaction involves the d_xz_ and d_yz_ orbitals with the π states of Gr, resulting in bonding π (d_xz_, d_yz_) and antibonding π* (d_xz_, d_yz_) states. The d_z2_ and d_xy_ orbitals, instead, remain almost unbound. When the underlying Ni(111) substrate is taken into account, its primary effect involves the broadening of the z‐component states (i.e. π/π* (d_xz_, d_yz_) and d_z2_) due to the coupling with the metallic states, leaving the d_xy_ as the only non‐bonding orbital.^[35]^ The filling degree and energy position of such a new set of bonding, antibonding, and non‐bonding states will depend on the specific transition metal atom, in particular here Co or Ni.

We computed and analyzed the density of states (DOS) for both the separate systems (CO + M@Gr/Ni) and upon interaction/bonding (CO^chem^‐M@Gr/Ni). Starting with the separate systems, in Co@Gr/Ni the orbital ordering for both spin channels is as follows: σ (d_x2‐y2_), π (d_xy_, d_yz_), d_xy_, d_z2_, π* (d_xy_, d_yz_), and σ* (d_x2‐y2_) (Figure 4b). In the spin‐up channel, π* (d_xy_, d_yz_) is below the Fermi level, while its counterpart in the spin‐down channel is empty. Both π (d_xy_, d_yz_) and π* (d_xy_, d_yz_) states can potentially participate in the backdonation to the ligand CO due to their appropriate symmetry. However, we expect that the π* (d_xy_, d_yz_) states will play a more significant role due to their higher energy level, closer to the CO LUMO orbitals (Figure 4b), which will favor the formation of a bond with the incoming CO molecule. Based on this, in the following analysis involving CO ligand binding, our focus will be mostly on the backbonding by the occupied π* (d_xy_, d_yz_) states.

In the case of Ni, due to the presence of one additional electron compared to Co, the π* (d_xy_, d_yz_) state in the spin‐down channel is below the Fermi level and thus occupied (see Figure 4c), leaving only the σ* empty. Unlike Co, the π* (d_xz_, d_yz_) states are now positioned deeper below the Fermi level. We expect that this difference might be relevant for explaining the distinct interaction of the two metals with CO. We mention, as observed from the slight asymmetry between the up and down states in Figure 4b,c, that in the case of Co some residual magnetization is present on the metal (0.57 μ_B_), while in the case of Ni the magnetization is nearly negligible (0.06 μ_B_), consistent with previous literature.^[35]^

**Figure 5:**
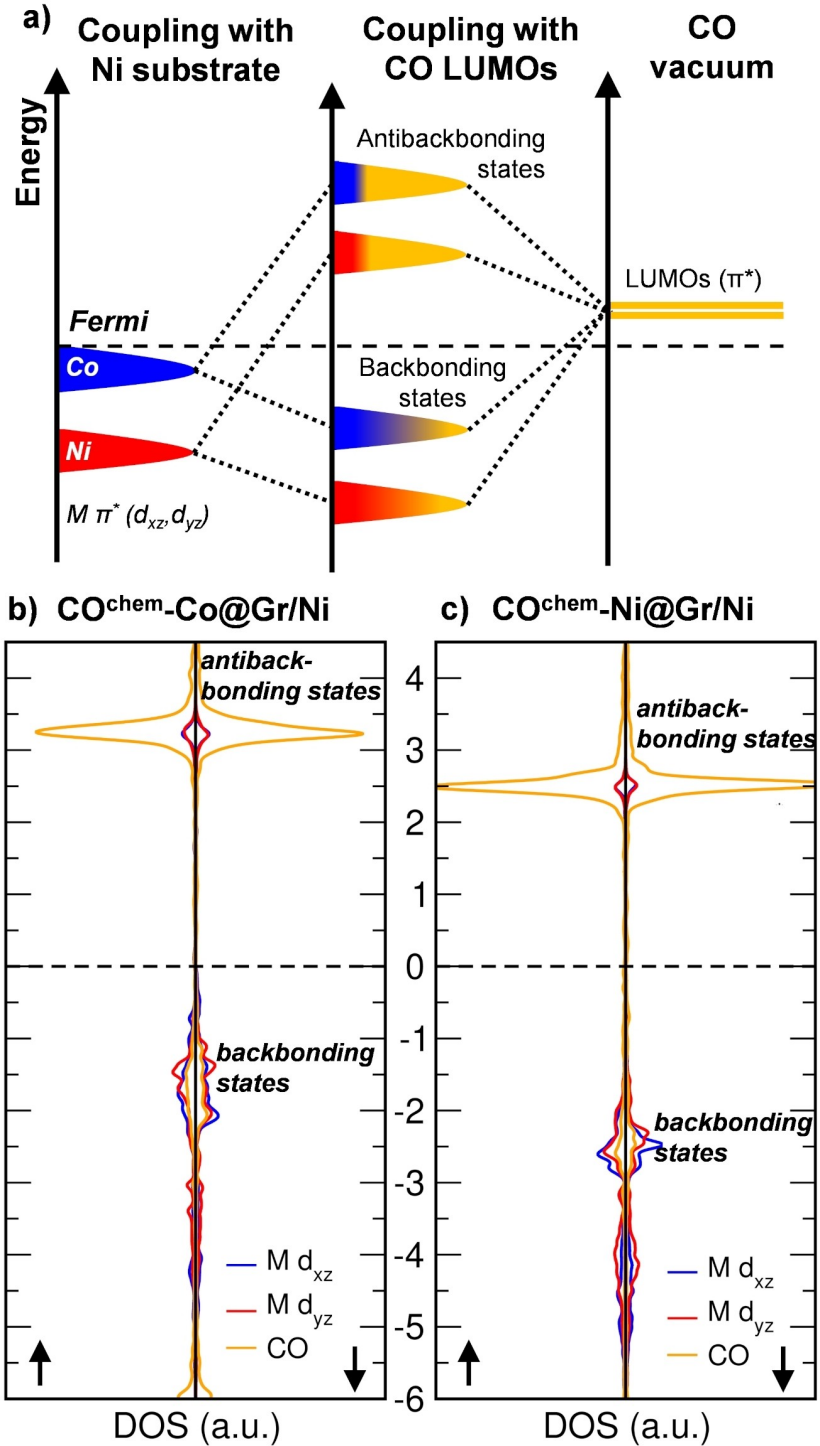
a) Schematic representation of the orbital interaction during CO chemisorption on the M@Gr/Ni system, where M represents either Ni (in violet) or Co (in cyan). For simplicity we considered spin‐up and spin‐down components together. The process begins with the coupling of M π* (originating from free‐standing M@Gr) with Ni substrate states, followed by the mixing with the empty LUMOs of the adsorbed CO molecule. This sequence results in the formation of a backbonding and antibackbonding state. b,c) Projected density of states (PDOS) on CO and metal π/π* states (in terms of d_xz_ and d_yz_ orbitals) for CO^chem^‐Co@Gr/Ni (b) and CO^chem^‐Ni@Gr/Ni (c) systems. The Fermi level is scaled to zero and is indicated by a dashed line. Up and down arrows indicate spin up and down projections, respectively.

Now that we have examined CO and the trapped metal atom individually, we proceed to investigate how they electronically interact during bonding. In Figure 5a, we present a schematic energy level diagram for CO adsorption on M@Gr/Ni, based on the computed projected density of states (PDOS) reported in Figure 5b. For both metals, we found that the π* (d_xz_, d_yz_) states can overlap with the CO LUMOs, lying higher in energy. As a result, new M–CO backbonding and antibackbonding states are formed, with the former being occupied and the latter empty, with a predominant metal and CO character, respectively, as evidenced by the PDOS in Figure 5b. The degree of overlap depends on two main factors: the correct symmetry and the difference in energy levels. In this scenario, the key difference between Co and Ni is the position of the π* (d_xz_, d_yz_) states, which are lower in energy for Ni (see Figure 4b,c). Consequently, the smaller energy gap between the CO π* LUMOs and the Co π* (d_xz_, d_yz_) orbitals allows for better mixing, explaining why CO binds more strongly to Co than Ni. This is further confirmed by a larger charge transfer to CO, which is 0.34 *e* from Co and 0.22 *e* from Ni. As a result of this electron transfer, the magnetization on Co is nearly quenched, dropping from 0.47 μ_B_ to −0.05 μ_B_.

To draw more general conclusions, we expanded our analysis to include Fe and Cu, positioned respectively before Co and after Ni in the periodic table. Interestingly, we found that both metals acquire the formal oxidation state of +1, similarly to Ni and Co (see Table S1). We attribute the resulting +1 charge on all types of trapped metal atoms to the fact that the hosting Gr layer is capable of accepting only one extra electron. Therefore, regardless of the specific metal considered, it is the Fermi level of the Gr/Ni(111) system that determines the extent of charge transfer.

In Figure S3, we compare the energy levels of the π (d_xz_,d_yz_)/π* (d_xz_,d_yz_) states (as determined by the PDOS on d_xz_ and d_yz_ orbitals) of Fe@Gr/Ni, Co@Gr/Ni, Ni@Gr/Ni, and Cu@Gr/Ni with the CO states of a CO molecule in the gas‐phase. We aligned the states of each system relative to the vacuum level to facilitate a direct comparison. Starting with Cu, the π* (d_xz_, d_yz_) states are completely filled and positioned deep in energy below the Fermi level, stemming from the d‐closed shell configuration of Cu^1+^ (3d^10^). As a consequence, the hybridization with the CO′s π* LUMO is smaller compared to previous metals, as confirmed by the lower binding energies and charge transfer to CO of −0.98 eV (Table S2) and 0.17 *e*, respectively. When moving to Fe, which has one electron less than Co, the π* (d_xz_, d_yz_) states are nearly unoccupied, featuring only a tiny part filled below the Fermi level. Consequently, the backbonding interaction with CO by π* (d_xz_, d_yz_) states is smaller compared to Co, as evidenced by the lower calculated binding energy (ΔE_bind_) of −2.44 eV (Table S2 and Figure 1). Details of the structural modification upon CO chemisorption, in terms of atomic distances, are reported in Table S3.

Upon this analysis, we found the following trend in terms of CO binding strength: Co > Fe > Ni > Cu and identified the π* (d_xz_, d_yz_) states energy level as a significant descriptor. While the surrounding environment of the confined metal does influence these states, as they originate from the mixing between d_xz_ and d_yz_ metal orbitals and Gr π band states (Figure 4a), it is ultimately the nature of the metal that dictates the chemical activity.

As a final aspect, we investigated how the Ni(111) substrate affects the adsorption of CO on the two confined metal atoms. We began by examining either a single Co or a single Ni atom confined within a free‐standing Gr monolayer (Co@Gr and Ni@Gr). The π* (d_xz_, d_yz_) states are unoccupied, in contrast with the corresponding Ni(111) supported cases, where electron donation from the underlying support filled them up (Figure S3b). Therefore, in the free‐standing systems, only the filled π (d_xz_, d_yz_) states, lying below the Fermi level, can provide backdonation to the CO molecule, as proven by the DOS of the chemisorbed CO on M@Gr (Figure S3b), where we observed the formation of backbonding and antibackbonding states for both metals (Figure S3c). As far as the effect of adsorption on the molecular stretching frequency (2131 cm^−1^), CO on Co@Gr exhibits a frequency of 1998 cm^−1^, while on Ni@Gr of 2039 cm^−1^. Notably, both values are higher (less red‐shifted) than those computed for CO adsorbed on Co and Ni embedded in Gr/Ni(111) (1964 cm^−1^ and 1996 cm^−1^, respectively), indicating a weaker M−CO interaction through backdonation in the unsupported cases. This is further reflected in the decrease in binding energies of 0.36 eV for Co and 0.38 eV for Ni going from a supported to an unsupported system. We attribute this to the emptiness of the π* (d_xz_, d_yz_) states in both Co@Gr and Ni@Gr, which makes them unable to participate in backbonding stabilization. Conversely, in the presence of a Ni(111) substrate, Gr becomes heavily n‐type doped,^[44]^ the π* (d_xz_, d_yz_) states become filled, allowing for an additional backdonation to CO. Therefore, we can assert that the underlying metal substrate plays a crucial role in determining the strength of the CO adsorption on single metal atoms embedded in Gr. This will impact the activation barrier of those chemical processes that involve the CO bond activation or cleavage.

The computational analysis presented above predicts that isolated Co atoms confined in Gr can stably adsorb CO at RT, whereas Ni cannot. This theoretical conclusion requires experimental validation. To this purpose, we build on the results of a recent study, in which we successfully synthesized a functionalized Gr layer on Ni via CVD growth^[31]^: this layer contains a controllable amount of Co single atoms alongside Ni single atoms, which are trapped in the Gr network during the growth process.^[33]^ We further demonstrated that, although both species appear as bright protrusions in the STM images, they can be readily distinguished by specific features. In the following, we present proof‐of‐concept investigations, where multiple techniques have been applied to this system, aiming at elucidating the chemical activity of Co and Ni single atoms toward CO adsorption.

**Figure 6:**
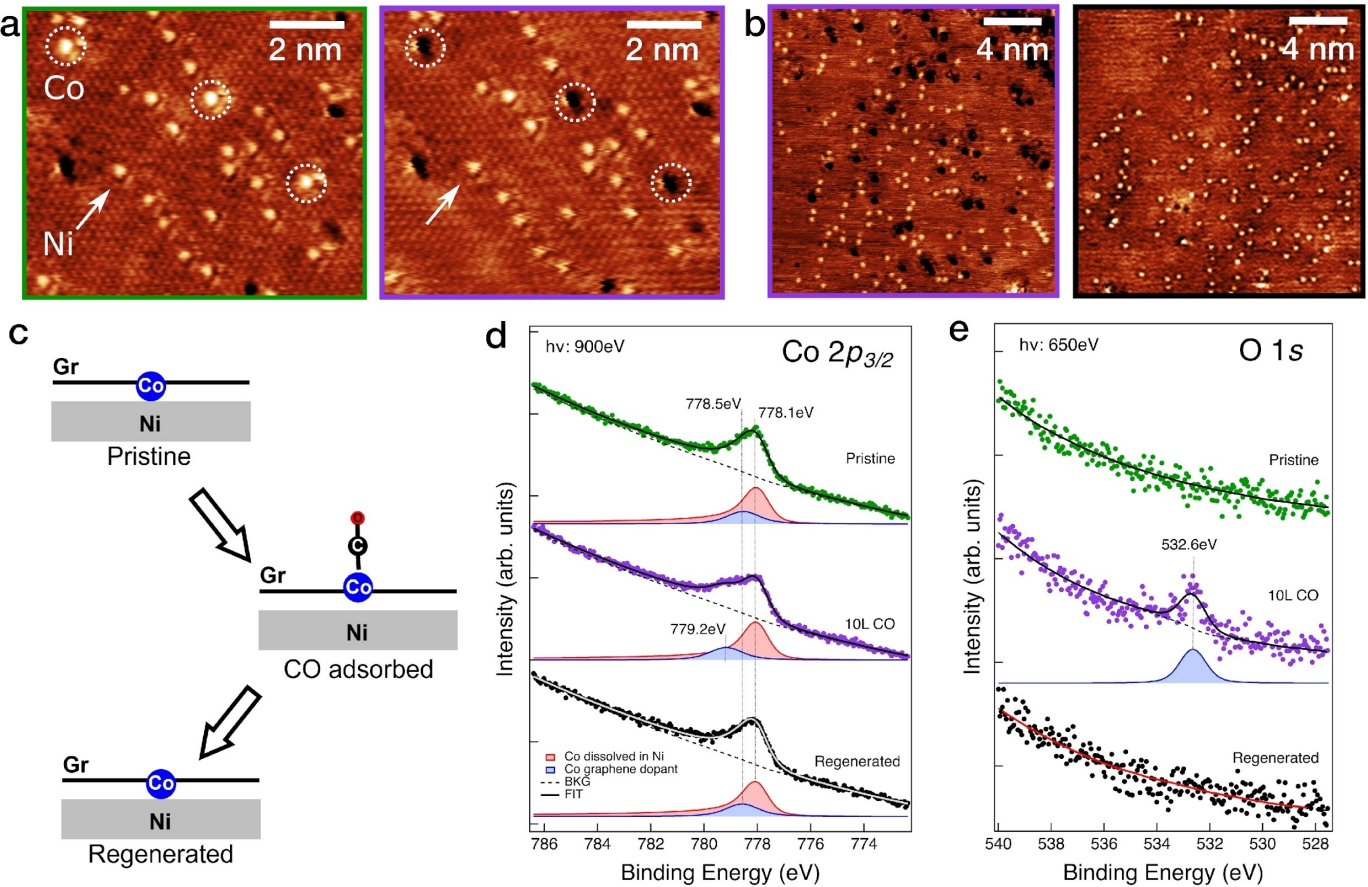
Experimental evidence of selective CO adsorption at Co single active sites. (a) STM images of Co and Ni atoms at room temperature before CO exposure (pristine, green) and after 3 minutes at 5 ⋅ 10^−7^ mbar CO (68 L, passivated, purple) in the same area. (b) Larger scale STM image after CO exposure (purple) and after regeneration by heating to 423 K (black). (c) Corresponding schematics of the pristine, passivated (CO adsorbed), and regenerated (after heat‐induced desorption of CO) states. (d) XPS spectra of Co 2*p*_*3/2*_ core level and (e) of O 1s core level recorded at the corresponding scenarios. Tunneling parameters: (a) I_T_ = 1.4 nA, U_T_= −0.1 V, (b, left) I_T_ = 0.5 nA, U_T_= −0.1 V, (b, right) I_T_ = 0.9 nA, U_T_ = −0.1 V.

Firstly, we applied STM and XPS to monitor the behavior of the Co and Ni co‐doped Gr layer upon CO gas exposure. Figure 6a shows high‐resolution STM images of the co‐doped layer at RT acquired before (left) and after (right) CO exposure, with Co and Ni dopants indicated. After in situ CO exposure at RT (3 min at 5 ⋅ 10^−7^ mbar, corresponding to 65 L (Langmuir)), exclusively Co dopants change their appearance, undergoing a contrast inversion from bright protrusions to dark depressions. The dark appearance of the adsorbate is typical for CO molecules adsorbed on metals.^[45]^ Therefore, we tentatively attribute the observed effect to the adsorption of a CO molecule on the Co sites, which become passivated. Entire regeneration of the passivated dopants is achieved by flash annealing the sample at 423 K, as shown in Figure 6b, where the passivated Co dopants (left) regained their bright appearance after thermal treatment (right). Notably, in agreement with the DFT calculations discussed above, no change in the Ni dopants is observed by STM throughout these steps. A corresponding representation of the process is displayed in Figure 6c, illustrating the pristine (before exposure), passivated (CO adsorbed), and regenerated (CO desorbed) layers.

To corroborate the STM results, we acquired XPS spectra of the pristine, exposed and regenerated co‐doped layers. Co 2*p*_*3/2*_ and O 1*s* core levels are presented in Figure 6d and 6e, respectively. The fitting procedure is described in detail in the SI. As previously reported,^[25]^ only a fraction of Co atoms are incorporated into the Gr layer: most of them either dissolve into the Ni bulk or intercalate at the interface between Gr and the Ni substrate, while formation of Co clusters on top of Gr can be excluded at this growth temperature.[^46^, ^47^] The pristine layer (green curves) exhibits a prominent asymmetric metallic Co 2*p*_*3/2*_ peak centered at about 778.0 eV (red component), arising from intercalated and dissolved Co, together with another symmetric component at slightly higher binding energy (BE) of 778.5 eV (blue component), which we assign to Co atoms trapped within Gr. The BE value of 778.0 eV corresponds to metallic Co, while the BE of 778.5 eV is shifted towards values that are found for compounds containing Co in a +2 oxidation state, i.e. typically around 780 eV.^[48]^ This indicates that embedded Co atoms present an oxidation state <+2, in agreement with the calculated positive atomic charge on Co of +0.80 *e*. Notably, no signal is detected in the O 1*s* core level region.

After passivation by CO (10 L, purple spectra), the Co *2p*_*3/2*_ metallic component does not change, while the entrapped Co component shifts to higher BE (778.5 → 779.2 eV), indicating an increase of the Co oxidation state, in agreement with the DFT calculated Bader charge variation of +0.22 *e* on the Co atom (+0.80 *e* → +1.02 *e*). In parallel, the O 1*s* peak at 532.6 eV appears, attributed to the adsorbed CO species. In the XPS experiment, exposure to 10 L of CO was sufficient to saturate the Co dopants (no change in the spectrum upon further dosing up to 60 L; see Figure S4). In contrast, a 65 L dose was required in STM to obtain complete passivation within the scanned area. This discrepancy can be primarily attributed to the shielding effect caused by the STM tip^[49]^ and to differences in the geometries of the two experimental setups; slight variations in Co coverage between the different sample preparations can also have a minor role regarding this issue.

The CO desorption upon annealing to 423 K, previously observed by STM, is further confirmed by the complete disappearance of the O 1*s* peak and by the shift to the pristine BE (778.5 eV) value of the Co *2p*_*3/2*_ component arising from entrapped Co (black spectra), indicating that the desorption temperature lies in the range from RT to 423 K.

We would like to emphasize that the passivation‐regeneration cycle illustrated in Figure 6c is entirely reversible. Upon further CO exposure on the regenerated surface, the same effect is observed as on the pristine surface‐passivation of the Co atoms by CO. This process has been repeated multiple times (at least three times) in STM, XPS, and TPD experiments, without any signs of structural changes or loss of active Co centers. As previously demonstrated, this system is highly robust, maintaining stability at temperatures of at least 700 K, even outside the vacuum system and in electrochemical environments.^[31]^ The CO atmosphere is no exception to this robustness. The reversibility and stability of CO adsorption provide a significant advantage over other single‐atom supports, such as the well‐studied Fe_3_O_4_(001), where adatom sintering has been observed upon CO exposure[^50^, ^51^].

**Figure 7:**
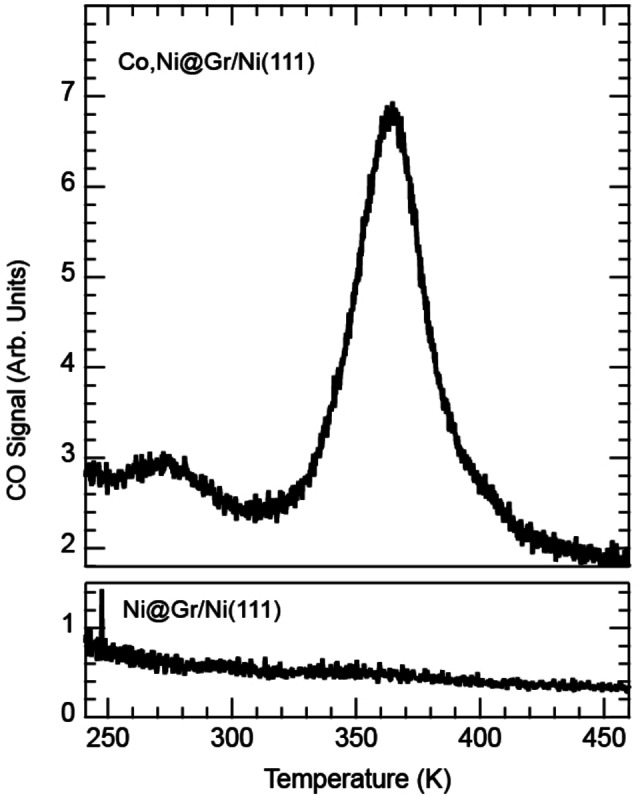
CO TPD experiment comparing the Co and Ni co‐doped Gr layer grown on Ni(111) (top panel) and only Ni doped Gr (bottom panel) without Co atoms.

Another direct confirmation of CO being adsorbed exclusively on Co and the precise temperature value of complete CO desorption have been obtained by highly sensitive temperature programmed desorption (TPD) analysis. Here, we investigated two differently doped Gr layers, in the same temperature range relevant for passivation/desorption identified in STM and XPS (namely from RT to 423 K): i) Co and Ni co‐doped Gr and ii) Ni‐only doped Gr on Ni(111). In Figure 7, the bottom panel, which corresponds to the Ni‐only doped Gr layer (i.e. without Co), shows no CO desorption in this temperature range, consistent with the STM experiments and DFT calculations, as discussed above. Conversely, the TPD curve of the Co and Ni co‐doped Gr, displayed in the top panel, remarkably shows a clear CO desorption peak at 365 K, occurring precisely in the temperature range in which the sample regeneration is observed by STM and XPS. By using the Redhead approximation for first‐order desorption,^[30]^ we estimate a desorption energy of 1.02 eV for CO on Co (setting the attempt rate to 10^13^ Hz and noting that its variation by one order of magnitude changes the desorption energy by 60 meV only), in fair agreement with our calculations (≈1.3 eV). The additional weak peak at 275 K, can be tentatively attributed to a small fraction (0.02 % ML) of Co dopants in sites with different atomic configurations.

Below 250 K, no feature can be attributed to the presence of Co (Figure S5). TPD experiments are therefore fully consistent with the STM and XPS measurements, confirming the absence of activity by the Ni dopants in this temperature range and a significantly different behavior of the two metals.

## Conclusion

In summary, our computational findings revealed a substantial difference in the adsorption free energy at RT of CO on Co with respect to Ni atoms, trapped in a C divacancy of Gr. To explain the differing behavior of these two metals when trapped in Gr, we have proposed an electronic descriptor based on the energy position of the π* (d_xz_, d_yz_) states. These π* (d_xz_, d_yz_) states are primarily involved in the interaction with CO through backdonation. Specifically, in the case of Co, the π* (d_xz_, d_yz_) states are positioned closer to the system‘s Fermi level, making them prone to back‐donate to CO, resulting in a stronger binding with Co than Ni.

As an experimental validation of our theoretical results, we prepared a Co and Ni co‐doped Gr layer on Ni(111) using a protocol we recently developed and exposed it to a CO at 5 ⋅ 10^−7^ mbar partial pressure. STM and XPS measurements, carried out before and after CO exposure at RT as well as after the regeneration of the metal sites by heating the Gr layer to 423 K, clearly demonstrate that CO binds exclusively to the Co site at RT, thereby confirming our theoretical predictions. The Gr layer without Co atoms finally excludes any activity of Ni atoms by showing no TPD CO desorption in the investigated temperature range.

In conclusion, this study, through the synergistic combination of DFT calculations with STM, XPS and TPD experiments, provides a deeper understanding of how the choice of single metal atoms may define reactivity through key electronic descriptors which may guide future catalyst design.

## Supporting Information

The Supporting Information includes a detailed description of the computational and experimental methodologies, ball‐and‐stick models showing the initial, intermediate, and final configurations along the CO adsorption path on Co@Gr/Ni as obtained through a CI‐NEB calculation (Figure S1), density of states projected onto the trapped metal d_xz_/d_yz_ states for various M@Gr/Ni systems (M=Fe, Co, Ni, and Cu) (Figure S2), optimized geometries and density of states for Ni@Gr and Co@Gr systems with and without chemisorbed CO (Figure S3), additional Co 2*p*_*3/2*_ and O 1*s* XPS spectra before and after CO exposure (Figure S4), CO TPD experiment comparing the Co‐doped Gr/Ni(111) layer and Gr/Ni(111) without Co atoms (Figure S5), Table S1 showing the computed atomic charge variations for the M@Gr/Ni systems, Table S2, which presents the energy decomposition analysis for CO adsorption on M@Gr/Ni systems, and Table S3 reporting the structural modification in terms of atomic distance M−C and M−Ni upon CO adsorption.

## Conflict of Interests

The authors declare no conflict of interest.

## Supporting information

As a service to our authors and readers, this journal provides supporting information supplied by the authors. Such materials are peer reviewed and may be re‐organized for online delivery, but are not copy‐edited or typeset. Technical support issues arising from supporting information (other than missing files) should be addressed to the authors.

Supporting Information

## Data Availability

Data needed to evaluate the conclusions in the paper are available at https://doi.org/10.5281/zenodo.13944534.
