# Supplementary material for: CO Adsorption on a Single‐Atom Catalyst Stably Embedded in Graphene
Source: Angew Chem Int Ed Engl. 2025 Jan 21;64(11):e202421757. doi: 10.1002/anie.202421757 (PMC11891640; doi:10.1002/anie.202421757)
Supplement: Supplementary file 1 — Supporting Information [file ANIE-64-e202421757-s001.pdf]

Angewandte Chemie
Eine Zeitschrift der Gesellschaft Deutscher Chemiker

Supporting Information

CO Adsorption on a Single-Atom Catalyst Stably Embedded in Graphene

D. Perilli, V. Chesnyak, A. Ugolotti, M. Panighel, S. Vigneri, F. Armillotta, P. Naderasli, M. Stredansky, M. Schied, P. Lacovig, S. Lizzit, C. Cepek, G. Comelli, H. Brune, C. Africh, C. Di Valentin**

SUPPORTING INFORMATION

CO Adsorption on a Single-Atom Catalyst Stably Embedded in Graphene

Daniele Perilli,[a,+] Valeria Chesnyak,[b,c,+,−] Aldo Ugolotti,[a] Mirco Panighel,[c,×] Stefano Vigneri,[b,c] Francesco Armillotta,[d] Pardis Naderasli,[d] Matus Stredansky,[c#] Monika Schied,[e,^] Paolo Lacovig,[e] Silvano Lizzit,[e] Cinzia Cepek,[c] Giovanni Comelli,[b,c] Harald Brune,[d] Cristina Africh,[c,*] and Cristiana Di Valentin[a,*]

[a] Department of Materials Science, University of Milano-Bicocca, via R. Cozzi 55, I-20125 Milano, Italy
[b] Physics Department, University of Trieste, via A. Valerio 2, Trieste 34127, Italy
[c] CNR - Istituto Officina dei Materiali (IOM), Trieste, Strada Statale 14, km 163.5, 34149 Trieste, Italy
[d] Institute of Physics, Ecole Polytechnique Fédérale de Lausanne (EPFL), Station 3, CH-1015 Lausanne, Switzerland.
[e] Elettra - Sincrotrone Trieste S.C.p.A., s.s. 14 km 163.5, 34149 Trieste, Italy
[+] D.P and V.C. equally contributed to this work
[−] Present address: School of Chemical, Biological, and Environmental Engineering, Oregon State University, Corvallis, OR, 97331, United States and Physical and Computational Sciences Directorate and Institute for Integrated Catalysis, Pacific Northwest National Laboratory, Richland, WA, 99354, United States.
[×] Present address: Scanning Probe Microscopy Laboratory, Department of Physics and Materials Science, University of Luxembourg, Luxembourg City L-1511, Luxembourg
[#] Present address: School of Chemistry, University of Birmingham Edgbaston, University Rd W, Birmingham B15 2TT, United Kingdom.
^ Present address: CNR - Istituto Officina dei Materiali (IOM), Trieste, Strada Statale 14, km 163.5, 34149 Trieste, Italy
[*] Corresponding authors: africh@iom.cnr.it, cristiana.divalentin@unimib.it

1. Computational and Experimental Methods

1.1. Computational Methods

DFT calculations were performed by means of the plane-wave-based Quantum ESPRESSO (QE) suite.[1,2] Ultrasoft pseudopotentials[3] were employed to describe the electron-ion interaction, treating Fe (3d, 4s), Co (3d, 4s), Ni (3d, 4s), Cu (3d, 4s), C (2s, 2p), and O (2s, 2p) as valence electrons. Energy cutoffs of 47 Ry and 326 Ry were chosen for kinetic energy and charge density expansion, respectively. To properly describe dispersive interactions, the van der Waals density functional vdW-DF2^{C09x},[4,5] was used for electron exchange-correlation. Spin polarization was included for all calculations.

We modelled the Gr/Ni(111) interface using a commensurate model where a (6×6) supercell of Gr was placed on top of a (6×6) supercell of Ni(111). To prevent interactions between neighboring images, we included a vacuum layer about 24 Å thick along with a dipole correction perpendicular to the surface. The Ni(111) surface was represented by a three-layer slab, wherein the bottom layer was fixed to bulk positions during geometry relaxation to simulate a semi-infinite solid. Geometry relaxation was performed at the Γ point. A denser k-points Monkhorst-Pack[6] meshes of 3×3×1 and 12×12×1 were used for the single-point and density of states calculation, respectively. The free energies (G) of the CO-chemisorbed structures (at T = 298.15 K) were computed following a similar method as we did for the H-adsorbed structures in our previous work.[7] The Climbing Image−Nudged Elastic Band (CI−NEB) method[8] was employed to simulate the CO chemisorption process, generating the minimum energy path of the reaction step and an evaluation of the energy barrier. Ball-and-stick models were rendered with the VESTA software.[9]

The free energies (G) of the intermediates (at T = 298.15 K) were computed by correcting the electronic energies (E) for the zero-point energy (ZPE), heat capacity (∫Cp dT), and the vibrational entropic term (S_{vib}), calculated as follows:

$$G = E + ZPE + \int C_p dT - TS_{vib}$$

Vibrational modes were calculated by performing a normal-mode analysis and all 3N degree of freedom of the adsorbates within the harmonic oscillator approximation. Corrections for the ZPE were included by calculating and diagonalizing the dynamical matrix in Γ point only. For the gas-phase CO molecule, the ideal gas limit approximation was employed, assuming a pressure of 1 bar.

1.2. Experimental Methods

All samples were synthesized according to Ref. 10 and corresponding cleaning procedures. The high Co evaporation flux method, leading to a higher Co coverage, was employed in the XPS and TPD experiments to maximize the Co signal. In the STM experiments the low flux method was sufficient to observe Co dopants and preferred to avoid crystal bulk contamination with Co.

STM measurements were performed with an Omicron variable-temperature scanning tunneling microscope (VT-STM) operated by a R9plus controller (RHK Technology) in constant current mode. STM images were processed utilizing standard operations of the Gwyddion software.[11] CO gas from a mini-can was dosed through a leak valve in the preparation chamber, that is connected to the preparation chamber, for in-situ investigation of the Co and Ni atoms in the same area via STM.

XPS experiments were conducted at the SuperESCA beamline at the Elettra synchrotron in Trieste. The same Ni(111) crystal and Co evaporator used in the STM experiments were mounted in the XPS setup to synthesize the Co and Ni doped Gr. Co 2p core level spectra were recorded at a photon energy of 900 eV and O 1s at 650 eV with energy resolution of ~200 meV and ~100 meV, respectively, in normal emission conditions and with the sample at RT. The intensity of each photoemission spectrum was normalized to the photon flux and the binding energy scale was referenced to the Fermi level of the substrate. Analysis of the data was conducted by performing a non-linear mean square fit, reproducing the photoemission intensity using Doniach–Šunjić line shape convoluted with a gaussian broadening, and a Shirley background. The Co $2p_{3/2}$ spectra have been fitted using two components, one corresponding to entrapped Co in graphene (not metallic, zero asymmetry) and another one due to dissolved and intercalated Cobalt (metallic, non-zero asymmetry). We used asymmetry and Lorentzian width gamma compatible with the values already known in literature,[12] while intensities, binding energies and gaussian widths were free parameters. The presence of two components in the pristine and regenerated spectra, where only an asymmetric photoemission peak is visible, is corroborated by the comparison between the pristine spectrum with that obtained after 10 L CO dose, as shown in fig. S3c: the total photoemission intensity is preserved, but there is a clear spectral weight change from the BE region centred at about 778.5 eV of the pristine spectrum to the BE region centred at about 779.0 eV of the spectrum after CO dosing (see main text for further details).

CO TPD dosing and detection are conducted in a separated UHV chamber hosted by the Laboratory of Nanostructures at Surfaces (LNS) group in Lausanne (EPFL). A home-built system has been used, where the sample surface is nearly entirely enclosed within a small desorption volume, separately pumped, enabling a detection limit as low as 3×10^{-5} ML/s.[13,14] A commercial Quadrupole

Mass Spectrometer (QMS, QMA 200 Pfeiffer Vacuum) is positioned at the end of this detection volume. The ionizer has been modified to prevent direct line of sight between the hot filament and the sample, while the internal walls of the detection volume are constructed from a chemically inert quartz tube. The probing gas (CO) is introduced directly into the Sniffer volume using electro-valves (Parker, series 99) activated by a rectangular voltage pulse of typically $1x10^{-4}$ μs duration and 28 V amplitude, corresponding to a partial opening of the valve.[14] The sample temperature was kept at 30 K during CO dosage and the TPD experiments reported in this manuscript are conducted with a heating rate of 1 K/s.

Supporting Images

Figure S1 illustrates the initial (CO^{phys}-Co@Gr/Ni), intermediate, and final (CO^{chem}-Co@Gr/Ni) configurations along the CO adsorption pathway on Co@Gr/Ni, as determined by a CI-NEB calculation. The transition of CO adsorption from the physisorbed to the chemisorbed state involves a gradual reorientation and shortening of the CO-metal bond. In the initial physisorbed state, CO interacts weakly with the metal center, characterized by a longer C-Co bond distance. As the system moves through the intermediate states, the CO molecule tilts and approaches the metal site, ultimately forming a stronger chemical bond in the chemisorbed state. A comparable pathway is observed for CO adsorption on Ni@Gr/Ni surface.

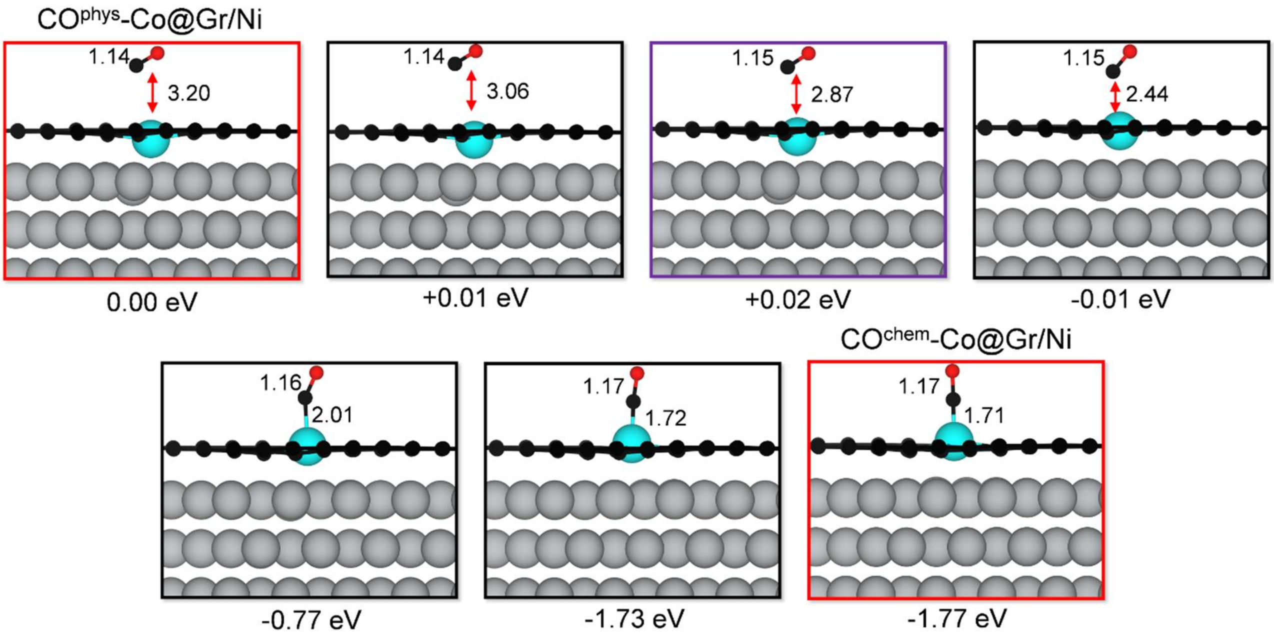

Figure S1. Ball-and-stick models (side view) showing the initial (CO^{phys}-Co@Gr/Ni), intermediate, and final (CO^{chem}-Co@Gr/Ni) configurations along the CO adsorption path on Co@Gr/Ni as obtained through a CI-NEB calculation. The C–O and C–Co distances (in Å) are provided for each configuration, along with the relative electronic energy (in eV) referenced to the initial configuration. Red and purple boxes highlight the initial and final configurations, respectively, while the highest-energy intermediate image is marked in purple.

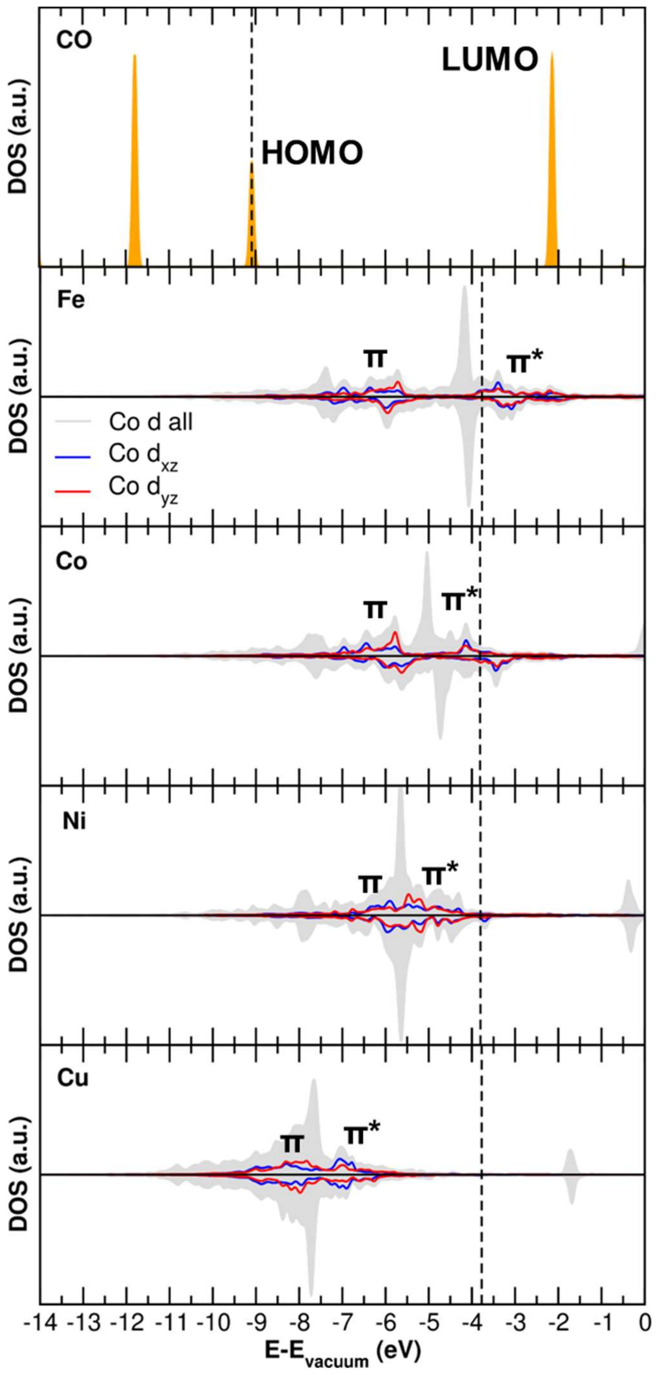

Figure S2. Projected (PDOS) density of states on metal d_{xz} and d_{yz} orbitals for M@Gr/Ni systems, with M representing Fe, Co, Ni, and Cu. Energy values are referred to the vacuum level, and the Fermi level is indicated by a dashed line. Each panel shows the spin up and spin down projections.

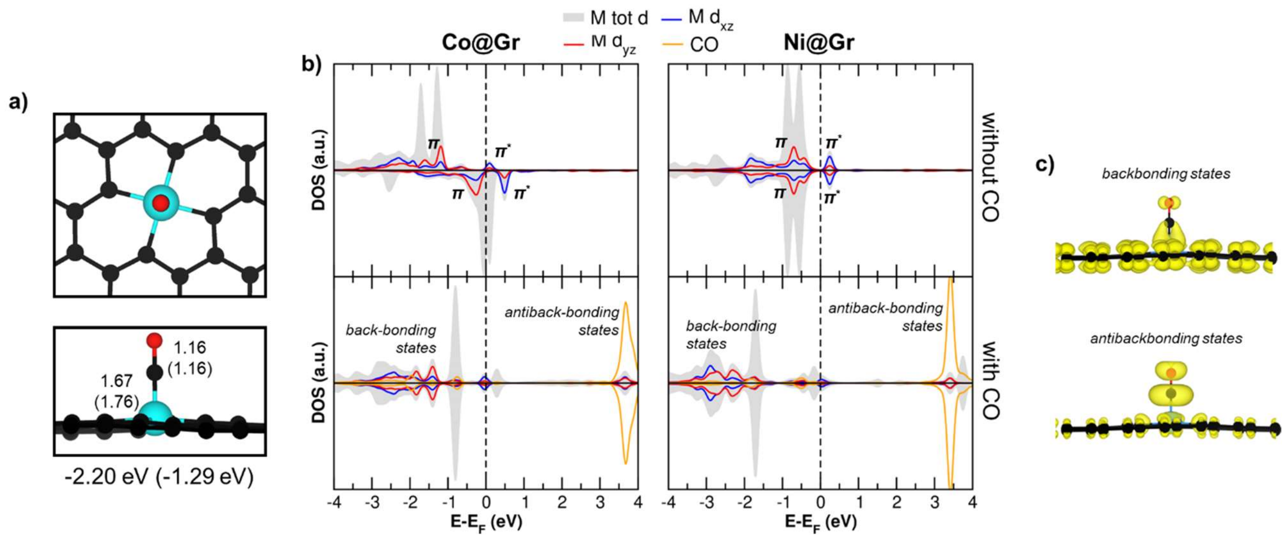

Figure S3. a) Top and side views of the optimized geometry for CO adsorbed on M@Gr. Color coding: C atoms in Gr are black, confined M atom is cyan, and C and O atoms in CO are black and red, respectively. Binding energies (ΔE_{bind}) and M-CO and C-O distances (in Å) for Co and Ni (in parentheses) are reported below the panel. b) Projected (PDOS) density of states on metal d_{xz} and d_{yz} and CO orbitals for M@Gr (top panels) and CO^{chem}/M@Gr (bottom panels) systems, with M = Co or Ni. Energy values are scaled with respect to the Fermi level, which is indicated by a dashed line. Each panel shows the spin up and spin down channels. c) Integrated local density of states (ILDOS) 3D plot for the backbonding and antibackbonding states of CO^{chem}/Co@Gr in panel (b). Isosurface value is 5×10^{-3} and 1×10^{-3} $e^-/bohr^3$ for the backbonding and antibackbonding states, respectively.

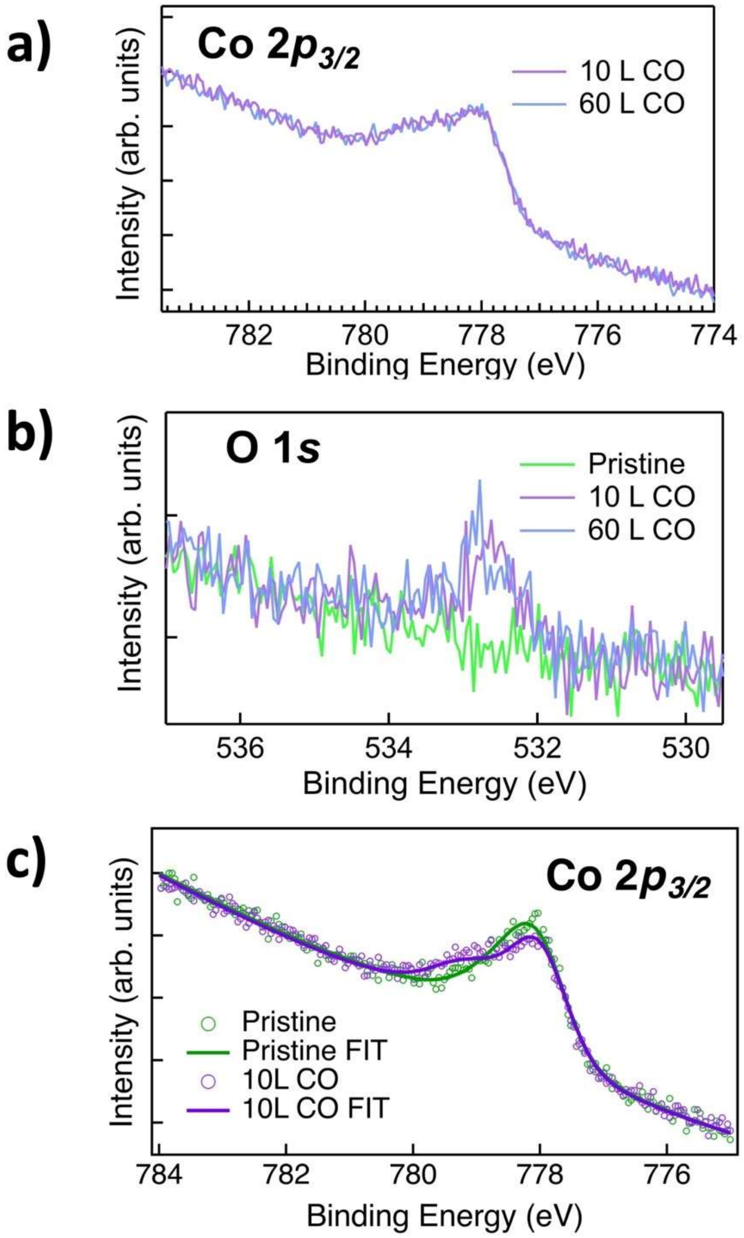

Figure S4. Superimposed XPS spectra for Co $2p_{3/2}$ (a) and for O 1s (b) recorded after 10 L and subsequent 60 L CO exposure (in purple and blue, respectively); (c) Comparison between Co $2p_{3/2}$ XPS spectra before (green) and after (purple) 10 L Co exposure. Lines correspond to fit results, open dots to experimental data.

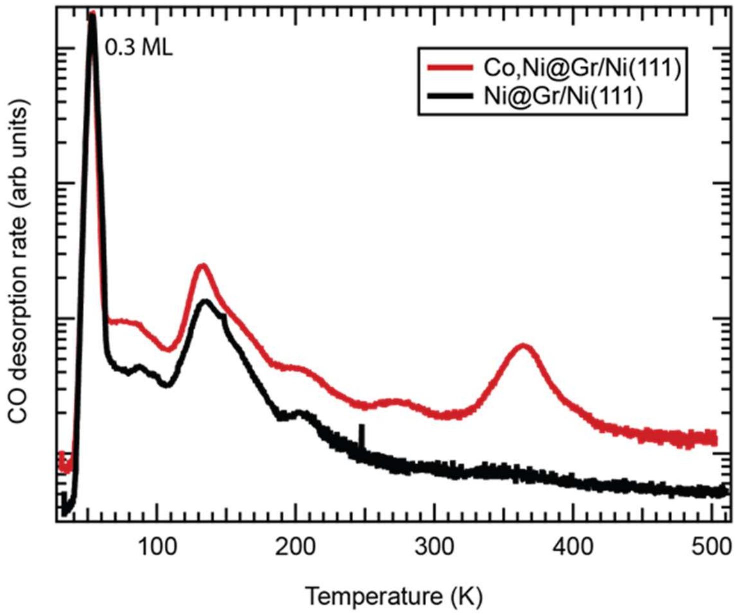

Figure S5. CO TPD experiment comparing the Co-doped Gr/Ni(111) layer (red curve) and Gr/Ni(111) without Co atoms (black curve).

Table S1. Changes in atomic charges (in *e*/C, *e*/Ni, and *e*/M) relative to the isolated atom, calculated using Bader analysis for Gr/Ni(111), Fe@, Co@, Ni@, and Cu@Gr/Ni(111) systems. Negative values indicate electron charge accumulation, while positive values indicate electron charge depletion. The second, third, and fourth columns report the charge variation for specific subgroups of atoms in each system: the Gr layer only, the Ni(111) substrate only, and the Fe/Co/Ni/Cu atoms trapped in Gr.

System	***Atomic charge (e)***		
	**Gr (*e* per C atom)**	**Ni(111) (*e* per Ni atom)**	**M (*e* per M atom)**
Gr/Ni(111)	-0.06	+0.04	-
Fe@Gr/Ni(111)	-0.08	+0.04	+1.02
Co@Gr/Ni(111)	-0.07	+0.04	+0.80
Ni@Gr/Ni(111)	-0.07	+0.04	+0.70
Cu@Gr/Ni(111)	-0.07	+0.04	+0.72

Table S2. Electronic energy (ΔE) contributions (as shown in **Table 1**) obtained from the energy decomposition analysis for CO chemisorption (*Chem*) on M@Gr, with M = Co and Ni. ΔG_{ads} values obtained at 298 K. All energy values are given in eV.

	Chem				
	$\Delta E_{def,M@Gr}$	$\Delta E_{def,CO}$	ΔE_{bind}	ΔE_{ads}	ΔG_{ads}
Co@Gr	+0.10	+0.03	-2.33	-2.20	1.51
Ni@Gr	+0.16	+0.02	-1.29	-1.11	-0.40

Table S3. Structural modifications in terms of bondlength upon CO chemisorption. Deformation energy cost for the M@Gr/Ni system upon CO chemisorption.

	**Fe@Gr/Ni**	**Co@Gr/Ni**	**Ni@Gr/Ni**	**Cu@Gr/Ni**
**M-C (Å)**	1.87 → 1.88	1.87 → 1.88	1.86 → 1.91	1.87 → 2.02/1.93
**M-Ni (Å)**	1.93 → 2.27	2.00 → 2.23	2.18 → 2.21	2.23 → 2.29
**$\Delta E_{def,M@Gr/Ni}$ (eV)**	+0.73	+0.59	+0.54	+0.63

References

[1] Giannozzi, P., Baroni, S., Bonini, N., Calandra, M., Car, R., Cavazzoni, C., ... & Wentzcovitch, R. M. (2009). QUANTUM ESPRESSO: a modular and open-source software project for quantum simulations of materials. Journal of physics: Condensed matter, 21(39), 395502.

[2] Giannozzi, P., Andreussi, O., Brumme, T., Bunau, O., Nardelli, M. B., Calandra, M., ... & Baroni, S. (2017). Advanced capabilities for materials modelling with Quantum ESPRESSO. Journal of physics: Condensed matter, 29(46), 465901.

[3] Dal Corso, A. (2014). Pseudopotentials periodic table: From H to Pu. Computational Materials Science, 95, 337-350.

[4] Lee, K., Murray, É. D., Kong, L., Lundqvist, B. I., & Langreth, D. C. (2010). Higher-accuracy van der Waals density functional. Physical Review B, 82(8), 081101.

[5] Hamada, I. (2014). van der Waals density functional made accurate. Physical Review B, 89(12), 121103.

[6] Pack, J. D., & Monkhorst, H. J. (1977). " Special points for Brillouin-zone integrations"—a reply. Physical Review B, 16(4), 1748.

[7] Perilli, D., Di Valentin, C., & Studt, F. (2020). Can single metal atoms trapped in defective h-BN/Cu (111) improve electrocatalysis of the H2 evolution reaction?. The Journal of Physical Chemistry C, 124(43), 23690-23698.

[8] Henkelman, G., Uberuaga, B. P., & Jónsson, H. (2000). A climbing image nudged elastic band method for finding saddle points and minimum energy paths. The Journal of chemical physics, 113(22), 9901-9904.

[9] Momma, K., & Izumi, F. (2011). VESTA 3 for three-dimensional visualization of crystal, volumetric and morphology data. Journal of applied crystallography, 44(6), 1272-1276.

[10] Chesnyak, V., Perilli, D., Panighel, M., Namar, A., Markevich, A., Bui, T.A., Ugolotti, A., Farooq, A., Stredansky, M., Kofler, C., Cepek, C., Comelli, G., Kotakoski, J., Di Valentin, C., Africh, C. (2024). Scalable Bottom-up Synthesis of Co-Ni-Doped Graphene. Science Advances just accepted.

[11] Nečas, D., & Klapetek, P. (2012). Gwyddion: an open-source software for SPM data analysis. Open Physics, 10(1), 181-188.

[12] Biesinger, M. C., Payne, B. P., Grosvenor, A. P., Lau, L. W., Gerson, A. R., & Smart, R. S. C. (2011). Resolving surface chemical states in XPS analysis of first row transition metals, oxides and hydroxides: Cr, Mn, Fe, Co and Ni. Applied Surface Science, 257(7), 2717-2730.

[13] Schlichting, H., & Menzel, D. (1993). Techniques for wide range, high resolution and precision, thermal desorption measurements: I. Principles of apparatus and operation. Surface science, 285(3), 209-218.

[14] Bonanni, S., Aït-Mansour, K., Hugentobler, M., Brune, H., & Harbich, W. (2011). An experimental setup combining a highly sensitive detector for reaction products with a mass-selected cluster source and a low-temperature STM for advanced nanocatalysis measurements. The European Physical Journal D, 63, 241-249.
